# Immunohistochemical staining for thyroid peroxidase (TPO) of needle core biopsies in the diagnosis of scintigraphically cold thyroid nodules

**DOI:** 10.1111/j.1365-2265.2007.03130.x

**Published:** 2008-06

**Authors:** U Yousaf, L H Christensen, Å K Rasmussen, F Jensen, C L Mollerup, J Kirkegaard, I Lausen, F Rank, U Feldt-Rasmussen

**Affiliations:** * Departments of Endocrinology, RigshospitaletCopenhagen, Denmark; † Departments of Pathology, RigshospitaletCopenhagen, Denmark; ‡ Departments of Radiology, Ultrasound Unit, RigshospitaletCopenhagen, Denmark; § Departments of Endocrine Surgery, RigshospitaletCopenhagen, Denmark; ¶ Otolaryngology – Head and Neck Surgery, RigshospitaletCopenhagen, Denmark

## Abstract

**Background:**

Cold thyroid nodules are common, in particular in iodine-deficient areas, but only a minority of them are malignant requiring surgery. Thyroid peroxidase (TPO) immunostaining of fine-needle aspiration cytology (FNAC) material has proven helpful in diagnosing cells from malignant lesions, but the procedure has its limitations in a routine setting.

**Purpose:**

To improve diagnosis and reduce surgery rate, the FNAC procedure was replaced by needle core biopsy (NCB), which was routinely stained for TPO by the monoclonal antibody mAb 47.

**Materials and methods:**

During a 5-year period 427 consecutive patients with a cold thyroid nodule were evaluated by ultrasound-guided NCB, which had been routinely stained for TPO in an automated immunostainer. Sensitivity and specificity and predictive values of the TPO immunostaining were estimated, based on the final diagnosis obtained from surgical resection.

**Results:**

The majority of nodules with benign NCB diagnosis were not surgically removed, and thus a subgroup of 140 operated nodules formed the basis for the calculations. Sensitivity and specificity for benign and malignant lesions were 100% if the oxyphilic variant of adenomas and minimally invasive follicular carcinomas were excluded. By inclusion of these, the values fell to 89% and 97%, respectively. The predictive value of a positive test was 96% and the predictive value of a negative test was 97%.

**Conclusion:**

TPO immunostaining was found to be a valuable adjunct to morphology in the diagnosis of cold thyroid nodules of the nonoxyphilic type.

## Introduction

Cold nodules of the thyroid gland present themselves as hypofunctioning areas by ^99,m^technetium scintigraphy. Until recently, Denmark was a country of iodine deficiency^1^ and therefore these nodules are common. The risk of malignancy varies from approximately 1% in the USA up to 24% in other countries depending on iodine intake and reference pattern.^1^–^6^ Most benign cold nodules in areas of iodine deficiency appear to be solitary nodules in a colloid goitre, and less frequently follicular adenomas or localized thyroiditis. Malignant nodules are mainly carcinomas, and, less frequently, lymphomas or metastases. In iodine-deficient areas papillary carcinoma is by far the most common of the malignant tumours (83·2%), followed by follicular/Hürtle-cell carcinoma (9·7%), medullary carcinoma (1·7%) and undifferentiated carcinoma (1%).^7^ However, the incidence of diagnosed thyroid carcinoma is increasing, with an estimated rise of 3% per year in both Denmark^8^ and the USA.^2^,^7^

Fine-needle aspiration cytology (FNAC) has been the standard method for differentiating between benign and malignant nodules, but as cystic degeneration, bleeding, necrosis and calcifications are common features of both benign and malignant nodules, routine FNAC is highly person dependent and may carry a low sensitivity and specificity, resulting in a high surgery rate for benign nodules.^9^–^14^ Other diagnostic methods have therefore been introduced, including ultrasound-guided FNAC^15^–^17^ and ultrasound-guided needle core biopsy (NCB).^18^–^24^ The enzyme thyroid peroxidase (TPO) appears to be changed early in malignant transformation, and it has been discussed whether this enzyme might be a marker of benign *vs.* malignant thyroid lesions.^25^ The monoclonal antibody 47 (mAb 47) has a high affinity for TPO of normal or benign thyrocytes but a very weak or absent affinity for the TPO of malignant epithelial cells, in particular those from nonfollicular malignancies.^26^–^30^

We have previously shown, in a prospective immunocytochemical study, that mAb 47 was a valuable tool for enhancing the ability to discriminate benign from malignant lesions in optimally obtained and handled FNACs. Sensitivity and specificity were 100% and 99%, respectively.^31^ In a routine setting, however, cytological sampling and immunostaining was not optimal with respect to suitable material, time and manpower, and we decided to replace ultrasound-guided FNAC with ultrasound-guided NCB as our standard sampling procedure.

## Materials and methods

A prospective study was conducted during the 5-year period from 1999 to 2004 with the purpose of evaluating the diagnostic sensitivity and specificity of ultrasound-guided NCBs routinely stained for morphological markers [haematoxylin/eosin (H&E), van Gieson/Alcian blue (VG/A)] and for TPO in an automated immunostainer. Three senior pathologists who specialize in diagnosing thyroid lesions participated. Ongoing mutual consultations secured diagnostic uniformity. The biopsies were given one of the following diagnoses: benign, not otherwise specified (NOS), colloid goitre, atypia, adenoma, suspicious, papillary carcinoma, undifferentiated carcinoma, and paraganglioma. The diagnosis ‘atypia’ was used if the tissue differed from normal but did not show any signs of malignancy, and ‘suspicious’ was used if a malignant diagnosis was assumed but was not obvious. The diagnosis ‘adenoma’ was short for the term ‘follicular neoplasia, unknown whether benign or malignant’. This was used particularly if the biopsy material contained part of a fibrous capsule. The NCB diagnosis based on routine H&E and VG/A morphology was compared with its TPO expression. The final diagnosis for the excised nodule was reached according to World Health Organization (WHO) guidelines from sections stained with H&E and VG/A, and, if necessary, by sections stained immunohistochemically for TPO, CK19, calcitonin, CD56 and thyroglobulin.^23^ All of these were compared with the TPO expression of the corresponding NCB.

TPO immunostaining was considered positive (benign) if 80% or more of the follicular epithelial cells were stained, either homogeneously in all cells or in a patchy pattern, and negative (malignant) if less than 80% of the cells were stained.^25^,^27^ The visual impression scoring was convincing to the degree where interobserver variations regarding TPO staining pattern (positive *vs.* negative) were not found.

All other participating doctors at the different diagnostic and therapeutic levels were also specialized in thyroid disease.

A total of 427 patients were included in the study: 334 women (aged 9–86 years, median 46 years) and 93 men (aged 15–85 years, median 50 years). Each patient had a palpable solitary thyroid nodule, which was cold by ^99,m^technetium scintigraphy, or a dominant cold nodule in a goitrous gland, for which they underwent, with local anaesthesia, ultrasound-guided NCB and TPO immunostaining. With regard to the sampling procedure, a preceding pilot study of 25 cases including both FNAC and NCB had already convinced the participating specialist at the ultrasound unit (F.J.) that the targeting was the same for the two procedures. Only sampling type and hence eligibility of the biopsy material differed. The study was consecutive and included all patients who were admitted to the hospital with a cold thyroid nodule. Only patients on thrombolytic treatment were excluded. Prior to sampling the patients were informed of the risk of slight bruising and pain at the biopsy site for a few days.

All patients were clinically and biochemically euthyroid. Three patients with insufficient biopsy material were excluded from the study, leaving a total of 424 patients.

### Surgery

Unilateral or bilateral thyroidectomy was performed in 140 patients (33%), while the majority of patients, 284 (67%), were not operated upon as a consequence of full diagnostic procedure including NCB diagnosis, patient's request or, in the case of undifferentiated carcinomas, inoperability. These patients were all enrolled in a regular follow-up programme including visits 7 days after sampling (histological diagnosis and possible side-effects of the procedure), followed up by regular visits every 6 months with clinical palpation, standard thyroid blood tests, and re-biopsy in case of any changes or irregularities. The last check-ups were registered after 2–7 years.

For the 140 surgically removed nodules, diagnostic sensitivity (% TPO-negative/malignant nodules) and diagnostic specificity (% positive/benign nodules) were calculated. We also calculated the predictive value of a positive test for malignancy (% TPO-negative malignant/TPO-negative biopsies) and the predictive value of a negative test (% TPO-positive benign/TPO-positive biopsies).

The study was part of an ongoing quality assurance programme. During the study period (and even today) NCB sampling was (and is) a routine procedure. All patients had been informed of and agreed to the procedure. The previous FNAC study with automatic inclusion of TPO staining and the pilot study comparing FNAC and NCB sampling had both been approved by the local ethical committee.

Possible changes in surgery rates for cold thyroid nodules diagnosed by FNAC and NCB, respectively, were evaluated by comparing lists of operated patients with pathology lists of all FNAC and/or frozen section biopsies during the previous 5-year period (1994–99).

## Results

**Fig. 1:**
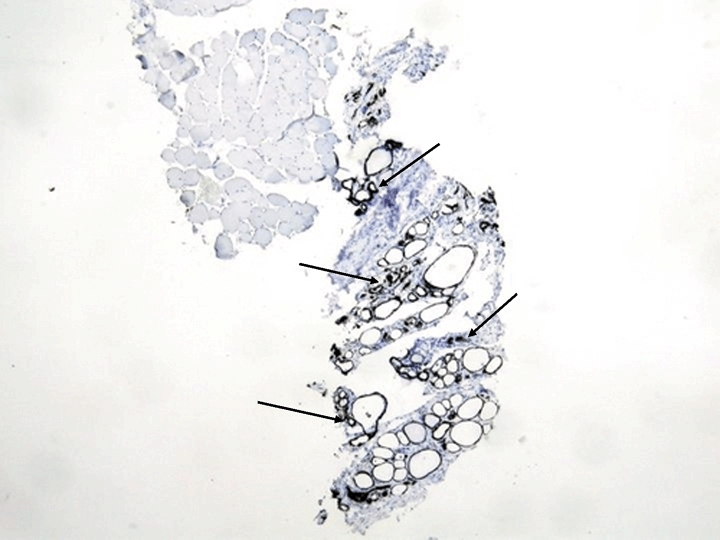
TPO stained typical NCB from a colloid goitre. All the follicular cells are intensely stained (arrows).

**Fig. 2:**
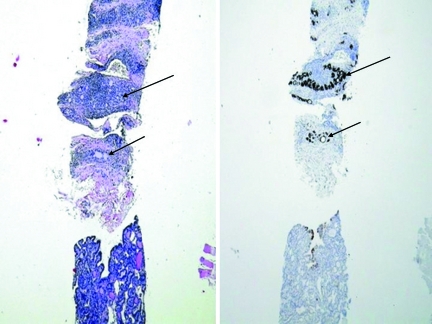
NCB from a papillary carcinoma, stained for H&E (left) and TPO (right). All tumour cells (bottom) are negative and all surrounding benign follicular cells are positive (arrows).

**Fig. 3:**
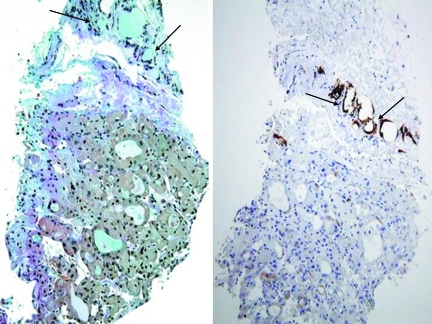
NCB from an oxyphilic adenoma, stained for Van Gieson Alcian blue (left) and TPO (right). Most oxyphilic cells fail to stain for TPO. The surrounding normal follicular cells are positive (arrows).

All follicular epithelial cells from benign, nonoxyphilic lesions showed an intense TPO-positive cytoplasmic staining (Fig. 1), while more than 80% of tumour cells of papillary, medullary, undifferentiated and widely invasive follicular carcinomas were TPO negative (Fig. 2). Table 1 shows the correlation between H&E morphology and TPO immunoreactivity on all 424 biopsies. One lesion turned out to be a paraganglioma of nonthyroid origin and hence TPO negative. This benign tumour was not subsequently removed. All the malignant, the one suspicious and two of the seven atypical lesions were TPO negative. Five biopsies with ‘atypia’ and eight with ‘adenoma’ contained oncocytic cells, which tended to give a spotted, irregular TPO staining (Fig. 3). Apart from slight bruising, which disappeared within a few days, no serious complications in the biopsy procedure (bleeding/haematoma, nerve lesion or pain lasting more than 1–2 days) were registered.

**Table 1:** Needle core-based diagnosis and corresponding thyroid peroxidase (TPO) expression of all (operated and nonoperated) cold nodules

H&E diagnosis	TPO expression		
	
	Positive	Negative	Total
Benign NOS	221		221
Goitre	139		139
Atypia	5	2	7
Adenoma	28	3	31
Suspicious		1	1
Papillary carcinoma		17	17
Undifferentiated carcinoma		7	7
Paraganglioma		1	1
Total	393	31	424

H&E, haematoxylin–eosin; NOS, not otherwise specified.

### No surgery

Biopsies from patients who did not undergo subsequent surgery were diagnosed as either ‘benign’ or ‘goitre’, and were TPO positive (Table 1). Included in this category also was a TPO-negative paraganglioma of nonthyroid origin and four cases of inoperable undifferentiated carcinoma, all of which were TPO negative. At follow-up none of the patients had undergone additional biopsy or surgery.

### Surgery

Table 2 shows the TPO staining pattern of the 140 surgically removed nodules. Diagnostic concordance between TPO-negative, NCB-based preliminary and postoperative final diagnosis was 100% for carcinomas of papillary (*n* = 17), widely invasive follicular (*n* = 1), medullary (*n* = 1) and undifferentiated (*n* = 3) subtypes (Table 2). Minimally invasive follicular carcinomas and adenomas were not reliably diagnosed from the NCB, neither by morphology nor by TPO immunostaining (Tables 2–4). On NCB, three had been diagnosed as oxyphilic adenomas being TPO positive (Table 4), and the remaining two had been diagnosed as TPO-negative papillary carcinomas (Table 3). The majority of adenomas were TPO positive (30/31), but for the minimally invasive follicular carcinomas three were TPO positive and two TPO negative (Table 2).

**Table 2:** Needle-core thyroid peroxidase (TPO) expression and final histological diagnosis for the 140 patients who underwent thyroidectomy

H&E diagnosis	TPO expression		
	
	Positive	Negative	Total
Benign NOS	15		15
Goitre	67		67
Adenoma	30	1	31
Papillary carcinoma		17	17
Minimally invasive follicular carcinoma	3	2	5
Widely invasive follicular carcinoma		1	1
Medullary carcinoma		1	1
Undifferentiated carcinoma		3	3
Total	115	25	140

NOS, not otherwise specified.

**Table 3:** Needle-core biopsy (NCB) diagnosis correlated to final histology diagnosis for thyroid peroxidase (TPO) negative excised cold nodules

NCB diagnosis	Final histology diagnosis						
	
	Adenoma	Papillary carcinoma	Minimally invasive follicular carcinoma	Widely invasive follicular carcinoma	Medullary carcinoma	Undifferentiated carcinoma	Total
Atypia		2					2
Adenoma	1		2				3
Suspicious		1					1
Papillary carcinoma		14		1	1	1	17
Undifferentiated carcinoma						2	2
Total	1	17	2	1	1	3	25

**Table 4:** Needle-core biopsy (NCB) diagnosis correlated to final histology diagnosis for thyroid peroxidase (TPO) positive excised cold nodules

NCB diagnosis	Final histology diagnosis				
	
	Benign	Goitre	Adenoma	Minimally invasive follicular carcinoma	Total
Benign NOS	10	37	9		56
Goitre	5	22	2		29
Atypia		1	2		3
Adenoma		7	17	3	27
Total	15	67	30	3	115

NOS, not otherwise specified.

In two cases of nodular goitre the final surgical specimen contained microscopic foci (2 mm and 3 mm, respectively) of papillary carcinoma (one in each patient). These were accidental findings, and the biopsy had not been taken from the area where they were identified.

The diagnostic sensitivity was 89% and the diagnostic specificity was 97%. The predictive values of a positive and a negative test were 96% and 97%, respectively.

Surgery lists and pathology reports from the previous 5 years (1994–99) revealed that 164 out of 303 cold nodules that had been diagnosed by FNAC alone (54%) had been removed surgically. In this study (1999–2004) only 140 out of the 427 cold nodules that had been diagnosed by NCB alone (33%) had been removed. Nodules removed during the pilot study and nodules diagnosed by frozen section sampling were not included.

## Discussion

This hospital-based, multidisciplinary prospective study running over a period of 5 years was carried out with the purpose of finding a more precise diagnostic procedure than FNAC in differentiating between benign and malignant cold thyroid nodules in an area with long-standing borderline low-incidence iodine intake.^1^,^3^,^5^

The aim was to reduce the number of unnecessary surgical thyroid resections, and TPO immunostaining, which had previously proven to be an excellent marker of benign *vs.* malignant lesions in optimally handled FNACs,^31^ was used systematically as an adjunct to morphology. The optimal approach that would have provided the added value of NCB would have been to prolong the pilot study, performing both NCB and FNAC in each case, and then offer surgery based on FNAC criteria. However, the pilot study had, in our hands, shown a more exact and reliable diagnosis from the NCB than from the FNAC procedure, and clinicians and patients decided in a substantial number of cases to refrain from surgery if a clear-cut benign diagnosis had been made in addition to the other results of the diagnostic work-up. As a result, only about one-third (33%) of the nodules (*n* = 140) were removed, apparently reducing surgery rate in comparison with the previous 5 years, where 54% had been removed, by 21%. However, this difference must be interpreted with caution, as both this and our previous FNAC study, which took place during 1993–96, may have instigated variations in sampling frequency. As previously noted by us^31^ and others,^28^,^30^ the encapsulated lesions, follicular adenomas and, in particular, minimally invasive follicular carcinomas, which in this study were of the oxyphilic type, were unreliable in their TPO staining and their sole responsibility for the value reductions (Tables 2–4). The majority of follicular adenomas were TPO positive (*n* = 30), as expected for benign lesions, and two out of the five minimally invasive follicular carcinomas were expectedly TPO negative. However, one adenoma with oxyphilic metaplasia was judged to be TPO negative, and three minimally invasive follicular carcinomas, also showing oxyphilic metaplasia, were judged to be TPO positive. This weakened the efficacy of TPO immunostaining for these particular lesions and it was therefore not possible to distinguish them preoperatively by their TPO expression alone. Currently, the only valid criteria for differentiating between adenoma and minimally invasive follicular carcinoma are focal capsular penetration and/or vessel invasion of the latter.^32^

Although the diagnostic sensitivity and specificity as well as predictive values for TPO positivity and negativity could only be established for one-third of the patients, the shift from FNAC to NCB allowed a greater number of pathologists to diagnose the lesions in a shorter time. It also provided both patients and surgeons with a more clear-cut benign diagnosis than before, and the frequency of thyroid surgery for cold nodules consequently appeared to be reduced by 21% when comparing surgery rate of the previous 5 years based on FNAC with the surgery rate of this study. For the same reason, specificity, sensitivity and predictive values of the TPO immunostaining was based on the 140 patients, who had a definite histological diagnosis after resection of their cold nodules.

Previous studies comparing FNAC and NCB have been conflicting regarding usefulness of the latter.^18^–^24^,^33^ Some have focused on a higher risk and the psychological impact of the NCB procedure,^20^ although bleeding was the only and rare complication observed,^18^,^19^,^24^ and others have found similar sensitivity and specificity values for the two procedures when optimally handled.^20^,^22^ However, a majority of reports have ended up recommending FNAC and NCB to be used complementarily,^19^,^21^–^24^ so that surgery on hyperplastic nodules^24^ and the number of false-positive and false-negative findings^22^ can be reduced to a minimum.

We are not aware of any previous prospective and systematic NCB studies with inclusion of TPO immunostaining as a routine procedure. However, other studies on surgical material have confirmed our observations. Although TPO was significantly reduced or negative in all nonfollicular neoplasms, it was not of sufficient discriminatory value in the diagnosis of follicular adenoma *vs.* carcinoma,^30^,^34^ in particular of the oxyphilic type.^34^ These results have been confirmed by observations on TPO mRNA analyses on 20 thyroid carcinomas,^28^ and it has further been demonstrated that the reduced TPO antigenicity is due to a quantitative rather than a qualitative loss.^29^

The reason to use a cut-off point for TPO positivity of 80% was based on observations on FNAC made by three other studies.^25^–^27^ The lesion was most likely to be benign if 80–100% of the epithelial cells were stained, and malignant if less than 80%, and in most cases, less than 40%, of the epithelial cells were stained. Sensitivity and specificity of this method were found to be 100% and 86·7%, respectively.^25^–^27^ In accordance with their findings, we did identify all nonfollicular carcinomas by their lack of TPO immunostaining. This was also true for the follicular variant of the papillary carcinoma. This tumour type can be difficult to diagnose on H&E morphology alone, but TPO immunostaining has in recent years been supplemented with immunostaining for the cytokeratin marker, CK19, a well-known reliable positive marker of papillary carcinomas.^35^

An obvious advantage of the NCB rather than the FNAC procedure is the ability to diagnose encapsuled lesions by the former, as part of the surrounding capsule will show up as a fibrous band on the biopsy.^36^ This has minimized the numbers of possible adenomas and therefore reduced the number of consequential surgical resections. A disadvantage of the NCB is its limited value in obtaining sufficient material from cystic or haemorrhagic lesions and the obvious psychological impact on the patient of the sampling procedure. Microscopic foci of papillary carcinoma were seen in two resections, although the benign TPO-positive biopsies had given morphological evidence of a benign goitre. This was not surprising. Papillary microcarcinomas, defined as tumours of 1 cm or less, are common incidental findings in a nodular goitre or in the thyroid glands of Graves’ disease.^37^,^38^ In this study they were 2 and 3 mm, respectively, and unlikely to have shown any clinical symptoms.

In conclusion, this study has shown that replacing FNAC with NCB sampling from the cold thyroid nodule has provided surgeons with a more specific diagnosis, and possibly a resultant reduced surgery rate. In addition, TPO immunostaining of an NCB is a valuable additional tool in the differentiation between benign and malignant scintigraphically cold thyroid nodules of the nonoxyphilic type.
